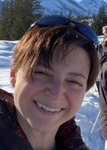# Looking Back: A Short History of the Discovery of Enzymes and How They Became Powerful Chemical Tools

**DOI:** 10.1002/cctc.202001107

**Published:** 2020-10-01

**Authors:** Christian M. Heckmann, Francesca Paradisi

**Affiliations:** ^1^ School of Chemistry University of Nottingham University Park Nottingham NG7 2RD UK; ^2^ Department of Chemistry and Biochemistry University of Bern Freiestrasse 3 3012 Bern Switzerland

**Keywords:** enzymes, protein engineering, history of biocatalysis, immobilization, green chemistry

## Abstract

Enzymatic approaches to challenges in chemical synthesis are increasingly popular and very attractive to industry given their green nature and high efficiency compared to traditional methods. In this historical review we highlight the developments across several fields that were necessary to create the modern field of biocatalysis, with enzyme engineering and directed evolution at its core. We exemplify the modular, incremental, and highly unpredictable nature of scientific discovery, driven by curiosity, and showcase the resulting examples of cutting‐edge enzymatic applications in industry.

## Graphical abstract

**In this historical** review we highlight the developments across several disciplines that were necessary to create the modern field of biocatalysis and showcase the resulting examples of cutting‐edge enzymatic applications in industry.

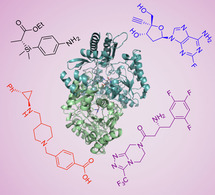

## 1 Introduction

**Figure 1:**
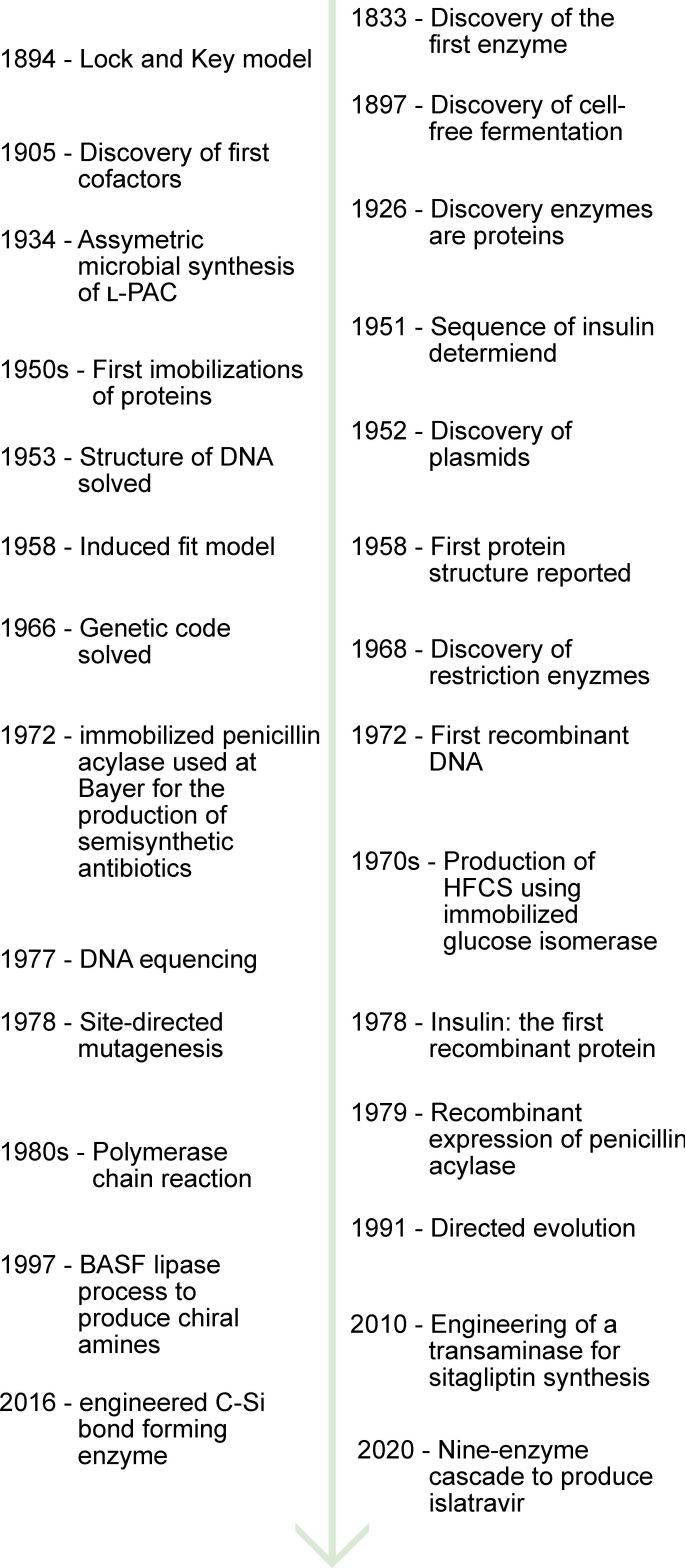
Timeline of major developments in enzymology, molecular biology, and biocatalysis.

Mesophilic organisms can carry out reactions under mild conditions, enabled by excellent catalysts: enzymes. Humans have, unknowingly at first, used these enzymes to their advantage for millennia, for example to ferment sugars into alcohol (as early as 7000 BC).^[1]^ So how did we get from biocatalysis being used unknowingly to the modern application at the forefront of chemical synthesis? In this review, the origin of modern biocatalysis is summarized (Figure 1), starting with the discovery of enzymes, and exploring the principles of biochemistry and molecular biology developed throughout the 20^th^ century. These stepping stones lead to the biotechnological advancements at the turn of the century which resulted in increasingly sophisticated applications of enzymes, in particular in industry. This review aims at condensing these biological developments primarily from the point of view of a chemist and is primarily intended to help chemists, but also scientists from other disciplines, entering the field, while also showcasing selected cutting‐edge applications of enzymes that may be of interest to biologists as well.

## 2 Early enzymology‐demystifying life

In 1833, diastase (a mixture of amylases) was the first enzyme to be discovered,^[2]^ quickly followed by other hydrolytic enzymes such as pepsin and invertase,^[3]^ but the term enzyme was only coined in 1877 by Wilhelm Kühne.^[4]^ The concept of catalysts, chemicals facilitating a reaction without undergoing any change themselves, was introduced in 1836^[5]^ by Berzelius who quickly hypothesized that enzymes were such catalysts.^[6]^ Yeast, which had been observed in ethanolic fermentations, was also viewed as a catalyst, but soon it was discovered that it was a living organism which at the time seemed to contradict that concept.^[7]^ Evidence from Pasteur that fermentation occurs in the absence of oxygen and failed attempts to isolate an enzyme able to carry out this transformation, were claimed as evidence by vitalists that a “life‐force” was necessary for these more complex transformations and that enzymes only carried out “simple” hydrolysis reactions.^[8a]^ Indeed, this is often framed as a dispute between Pasteur and Liebig, with the former supporting vitalism and the latter supporting a mechanistic view that ascribes no special place to life. However, it appears more accurate to say that Pasteur supported the idea that fermentation was carried out by yeast through chemical means whereas Liebig opposed the idea of any causal link between yeast as a living organism and the catalytic fermentation reaction, instead thinking that the decay of yeast in the presence of oxygen was catalyzing the formation of alcohol from sugar.^[8]^ Finally, in 1897, Eduard Büchner showed that a dead yeast extract could carry out the same fermentation reaction as living yeast, thus dealing the final blow to vitalism, which had already been on the decline (Nobel Prize in Chemistry 1907).[^3^, ^8a^]

The fermentation of sugars into ethanol and carbon dioxide was attributed to “zymase.” Further investigations started to reveal reaction intermediates, and dependency on phosphate and “co‐zymase” (A. Harden and, H. von Euler‐Chelpin; Nobel Prize in Chemistry 1929) and started to untangle glycolysis. However, the chemical nature of enzymes was still being debated. In 1926, James B. Sumner crystallized the first enzyme (urease), and confirmed it was a protein.^[9]^ John H. Northrop also crystallized several other proteins, amongst them pepsin, trypsin, and chymotrypsin.^[6]^ They were awarded the Nobel Prize in Chemistry in 1946; in his Nobel lecture,^[9]^ Sumner remarks that

“The organic chemist has never been able to synthesize cane sugar, but by using enzymes, the biological chemist can synthesize not only cane sugar but also gum dextran, gum levan, starch and glycogen.”

**Scheme 1:**
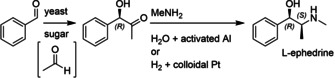
Synthesis of L‐ephedrine: Benzoin‐type addition of acetaldehyde (formed *in‐situ* by yeast metabolism) to benzaldehyde (now known to be catalyzed by pyruvate decarboxylase),^[14]^ followed by reductive amination with methylamine.^[11]^

Indeed, a whole range of industrial applications of mainly whole organisms but also some enzyme preparations had already been developed. For example, glycerol (used in the production of explosives) was produced on a 1000 ton per month scale in Germany during world war I, employing fermentation in yeast with the final acetaldehyde reduction step inhibited by sulfite, resulting in dihydroxyacetone phosphate reduction. By 1949, citric acid was almost exclusively produced using the fungus *Aspergillus niger* (ca. 26,000,000 pounds per year in the US alone), even though it was not understood how the organism produced it.^[10]^ In 1934, a patent was granted for the condensation of acetaldehyde (produced *in‐situ* from glucose) with benzaldehyde catalyzed by whole yeast, giving l‐phenylacetylcarbinol, which was then further reacted to give l‐ephedrine, a stimulant used during anesthesia, as a decongestant, and also a pre‐cursor to illicit drugs such as methamphetamine (Scheme 1).^[11]^ This procedure is still used today, highlighting the power of an efficient biocatalytic process.^[12]^ Enzymes prepared from fungi or bacteria became alternatives to those initially obtained from plants or animals (e. g. amylases and proteases). Purified proteases were used to clarify beer since 1911, pectinases (from various fungi or malt) were used to clarify juices and wine.^[9]^ In the early 1950s, several species of fungi were applied to the regio‐selective hydroxylation of steroids for the production of cortisone, which had been impossible using chemical means.^[13]^

**Figure 2:**
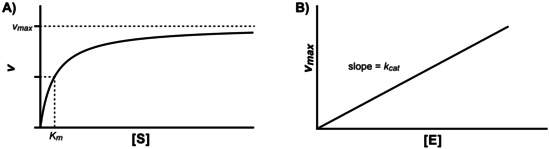
Plots of the Michaelis‐Menten model, illustrating equation 1. A) Velocity vs. substrate concentration under constant enzyme concentration. The substrate affinity *K_m_* corresponds to the substrate concentration at which the reaction reaches half of *v_max_*, which is the velocity at infinite substrate concentration. B) *v_max_* vs. enzyme concentration. The reaction is first‐order with respect to enzyme concentration, with the rate constant *k_cat_*.

By 1949 a vast number of enzyme classes had been discovered and characterized extensively. Many pathways and intermediates were fully uncovered, yet little was known with regard to the mechanism by which individual enzymes worked.^[9]^ Through the famous lock‐and‐key model, proposed by Emil Fischer in 1894,^[15]^ as well as the Michaelis‐Menten model of enzyme kinetics from 1913 (Equation 1, Figure 2)^[16]^ it was understood that a substrate has to bind to the enzyme prior to catalysis, yet how this binding proceeds and how catalysis occurs afterwards was unsolved. The ratio of *k_cat_/k_uncat_* has been found to be as high as 10^17^, crowning enzymes as exceptional catalysts. Interestingly, most enzymes have similar *k_cat_* values (within two orders of magnitude), while the rate constants of the corresponding un‐catalyzed reactions (*k_uncat_*) vary wildly.^[17]^

$$a)\;\;\;E+S\;\vec{\leftarrow }ES\to E+P\;b)\;\;\;v=\frac{v_{max}\left[S\right]}{K_{m}+\left[S\right]}=\frac{k_{cat}{\left[E\right]}_{0}\left[S\right]}{K_{m}+\left[S\right]}$$

Equation 1: The Michaelis‐Menten model and equation, in modern form: a) Enzyme and substrate combine in a reversible fashion to form the enzyme‐substrate complex, which then goes on to release the enzyme and product in an irreversible reaction. b) Under the assumption of a steady state concentration of the enzyme‐substrate complex, the Michaelis‐Menten equation can be written, describing the consumption of substrate depending on substrate concentration [S], maximum velocity *v_max_* (itself dependent on enzyme concentration [E]_0_), and substrate affinity *K_m_*.

## 3 Enzyme structures and elucidation of mechanisms

In 1948 Linus Pauling proposed that enzymes had to stabilize the transition state rather than the substrate as proposed by Fischer.^[18]^ The detailed concept of a transition state itself had only been developed less than two decades earlier.^[19]^ This was further refined by Koshland in 1958,^[20]^ proposing the concept of “induced‐fit,” explaining the specificity of enzymes on an abstract level. In parallel to this abstract understanding, a more detailed understanding of the structure of proteins was being developed. It had been hypothesized since the beginning of the 20^th^ century that proteins were composed of chains of amino acids connected via amide bonds;^[21]^ however, the order or even the relative amount of amino acids was not well understood, and indeed the peptide hypothesis itself was frequently questioned.^[22]^ In 1951, Sanger determined the amino acid sequence (referred to as primary structure) of insulin, revealing that indeed as expected it was a well‐defined sequence of amino acids linked by amide bonds.^[23]^ He received his first Nobel Prize in Chemistry in 1958 for this work.

**Figure 3:**
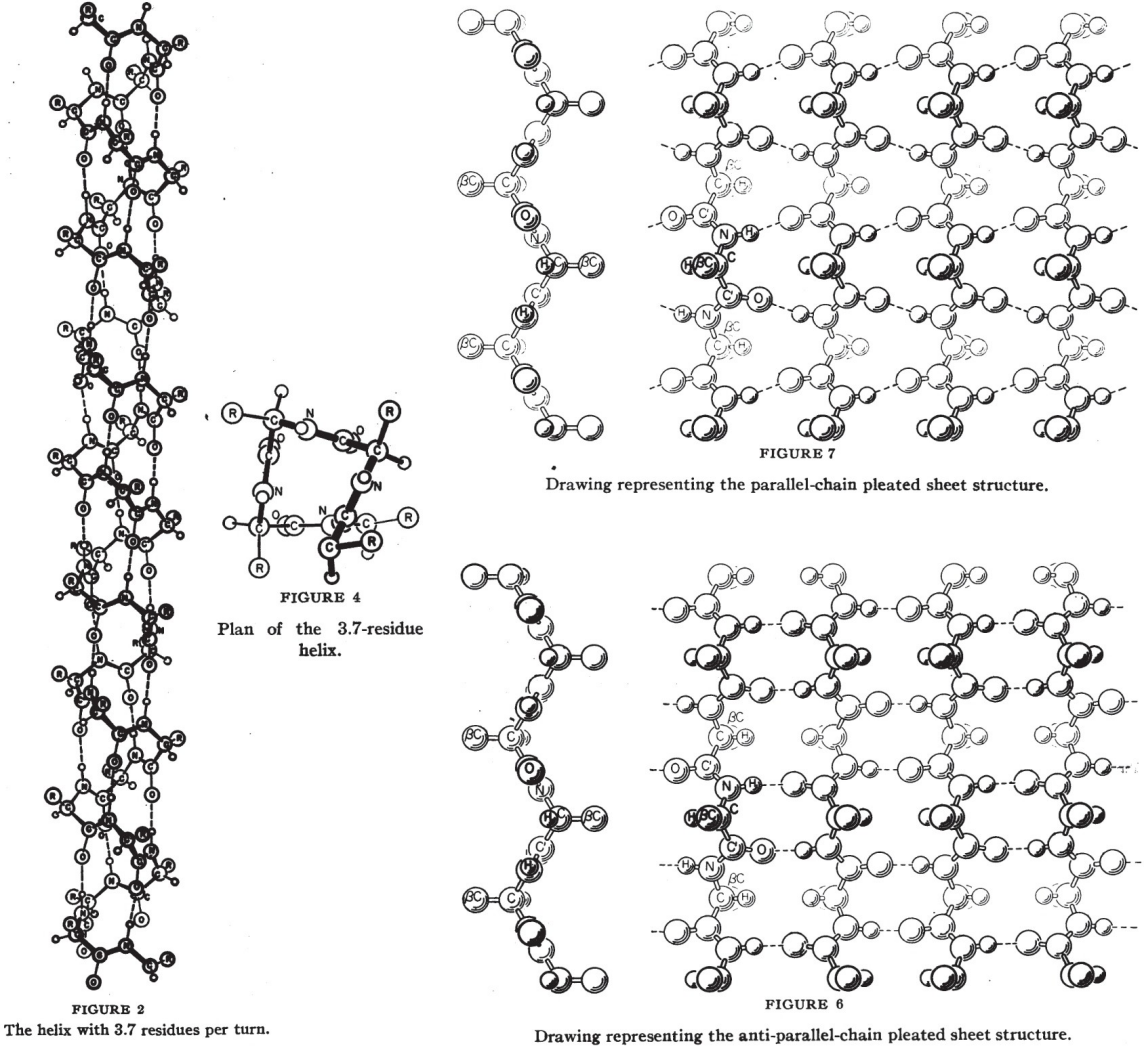
The original drawings of an α‐helix (left) and parallel and anti‐parallel β‐sheets (right), published by Pauling in 1951.[^24b^, ^24d^]

Famously, Linus Pauling proposed how a chain of amino acids might fold into regular geometric features (i. e. α‐helices and β‐sheets; referred to as secondary structure, Figure 3) while sick in bed, based on his detailed understanding of the rigidity of the amide bond and “reasonable” interatomic distances. The rigorous understanding of chemical bonds had just been developed for which Pauling received his Nobel Prize in Chemistry in 1954.^[24]^

**Figure 4:**
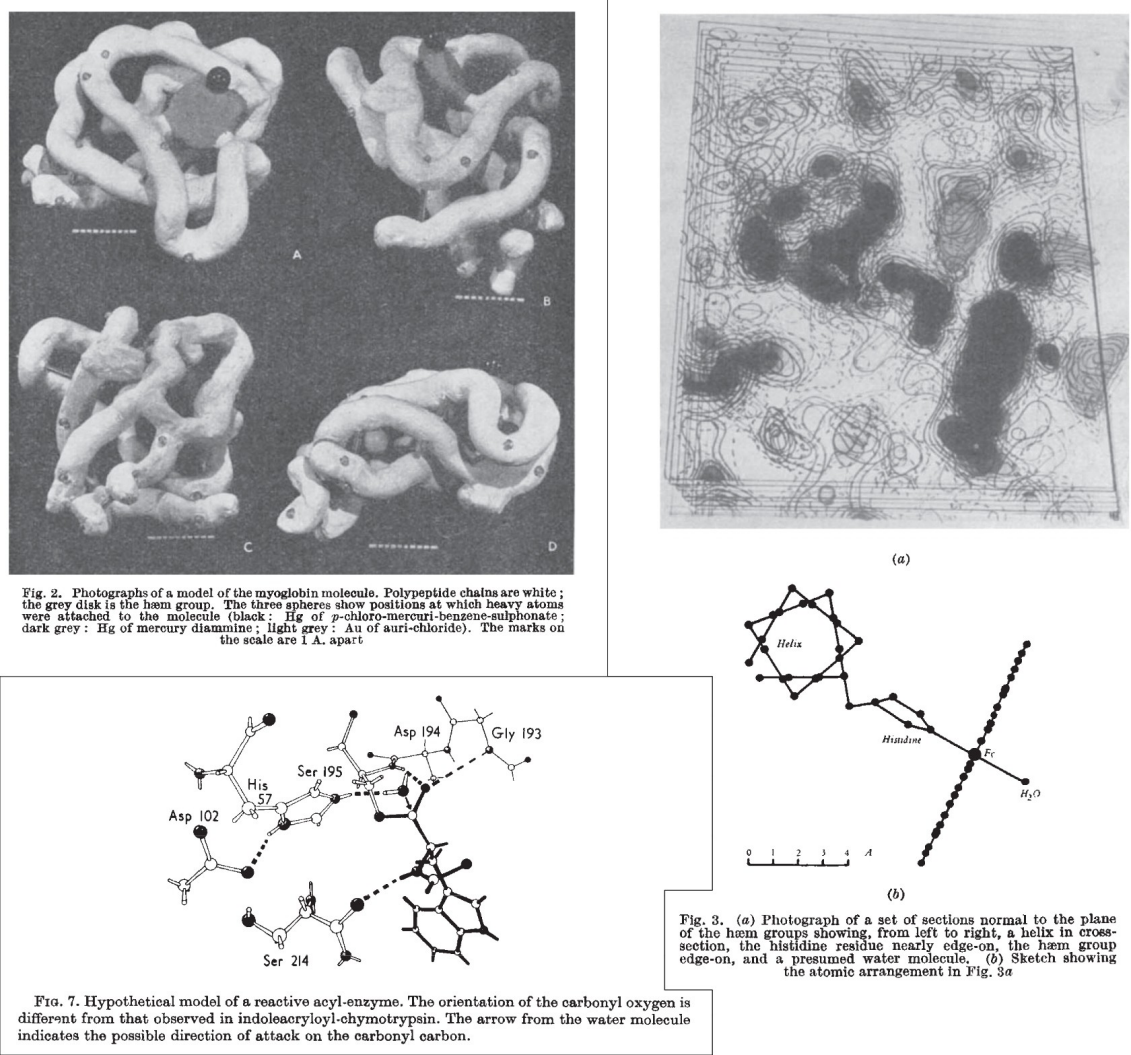
Top left: Clay model of the first X‐ray structure of a protein, myoglobin, at 6 Å resolution.^[26a]^ Right: electron density sections of myoglobin at 2 Å resolution and sketch of groups coordinated to iron.^[26b]^ Bottom left: Model of the catalytic triad and oxyanion hole of chymotrypsin, as inferred from crystal‐structures.^[31c]^

In the meantime, X‐ray crystallography, developed from 1912 onward by Max von Laue, (Nobel Prize in Physics in 1914) and William and Lawrence Bragg (Nobel Prize in Physics in 1915), had become more sophisticated and was being applied to increasingly complex compounds. Evidence for Pauling's secondary structure from X‐ray diffraction was reported by Max Perutz in 1951.^[25]^ The first structures of proteins were solved in 1958–1960 by John Kendrew and Max Perutz (Nobel Prize in Chemistry in 1962).^[26]^ This was initially met with some degree of disappointment as it revealed that proteins were “messy” (Figure 4) and squashed the hope that solving the structure of one protein would reveal the structure of all proteins (in contrast to DNA where that expectation largely held true).^[27]^ However, as higher resolution structures were obtained the insight that could be gained into the mystery world of enzymes became apparent and many groups set forth to investigate not just proteins but enzymes (Figure 4).^[28]^

Structures of lysozyme were solved in 1965,^[29]^ and included structures of the enzyme with inhibitors bound to it, revealing the location and residues of its active site. Other enzyme structures solved around this time include bovine carboxypeptidase A in 1967,^[30]^ both with and without substrate bound‐revealing conformational changes (in agreement with the induced fit hypothesis) as well as key interactions between substrate and enzyme. The crystal structure of chymotrypsin (also in 1967)^[31]^ paved the way to uncover the classic catalytic triad and oxyanion hole of proteases (as well as esterases and other hydrolytic enzymes; Figure 4). In 1971, the Protein Data Bank (PDB) was founded with seven structures,^[28]^ reaching 50 structures in 1979, and 100 structures three years later. At the end of 2019 it contained almost 160,000 structures.^[32]^

In parallel to the increasing understanding of the structure of enzymes, newly developed physical‐chemistry techniques were also employed to elucidate mechanisms, such as detailed kinetics, isotopic labelling, isotope effects, and spectroscopic techniques.^[33]^ The first mechanisms to be elucidated in that way were of enzymes employing co‐enzymes, as the structures (fragments) of co‐enzymes were determined before the structures of whole proteins. Indeed, as early as 1936,^[34]^ Otto Warburg showed that certain pyridines (analogous to nicotinamide that could be obtained from hydrolysis “co‐zymase”) could transfer hydrides reversibly, implying that such a hydride transfer plays a role during glycolysis (he had previously received the Nobel Prize in Physiology or Medicine in 1931 for his work on the role of iron in respiration).

**Figure 5:**
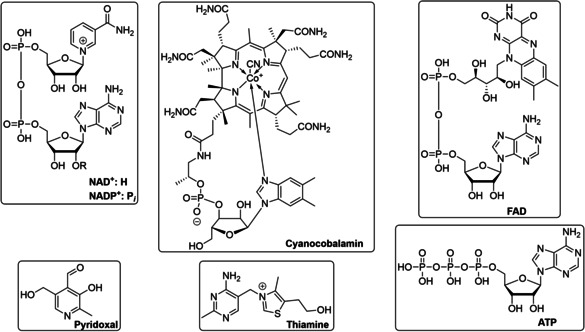
Selected structures of common cofactors that were known by 1955: NAD^+^, NADP^+^ and FAD are redox catalysts, ATP transfers energy released during glycolysis, thiamine is the co‐factor of pyruvate decarboxylase during fermentation, and pyridoxal is the co‐factor of transaminases, which are of particular industrial importance, as well as racemases, decarboxylases, and lysases involved in amino acid metabolism.

The full structure of thiamine (cocarboxylase) had been proved in 1936 by Williams and Cline.^[35]^ The structures of pyridoxine, as well as of biologically relevant derivatives pyridoxal and pyridoxamine were established in the early 1940s by Esmond Snell soon after the discovery of transaminases.^[36]^ Full structures of NAD(P)(H) (co‐zymase),^[37]^ ATP,^[38]^ and FAD^[39]^ were proved by Alexander Todd in the late 1940s and 50s (Nobel Prize in Chemistry in 1957). The structure of Vitamin B_12_ (cyanocobalamin) was solved through X‐ray crystallography by Dorothy Hodgkin in 1955^[40]^ (Figure 5; Nobel Prize in Chemistry in 1964).

**Figure 6:**
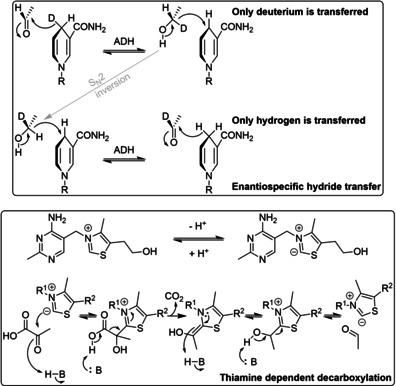
Selected mechanisms of co‐factors that were being elucidated in the 1950s: enantiospecificity during hydride transfer from NAD(P)H in alcohol dehydrogenases, and thiamine‐dependent decarboxylation.^[33b]^

**Figure 7:**
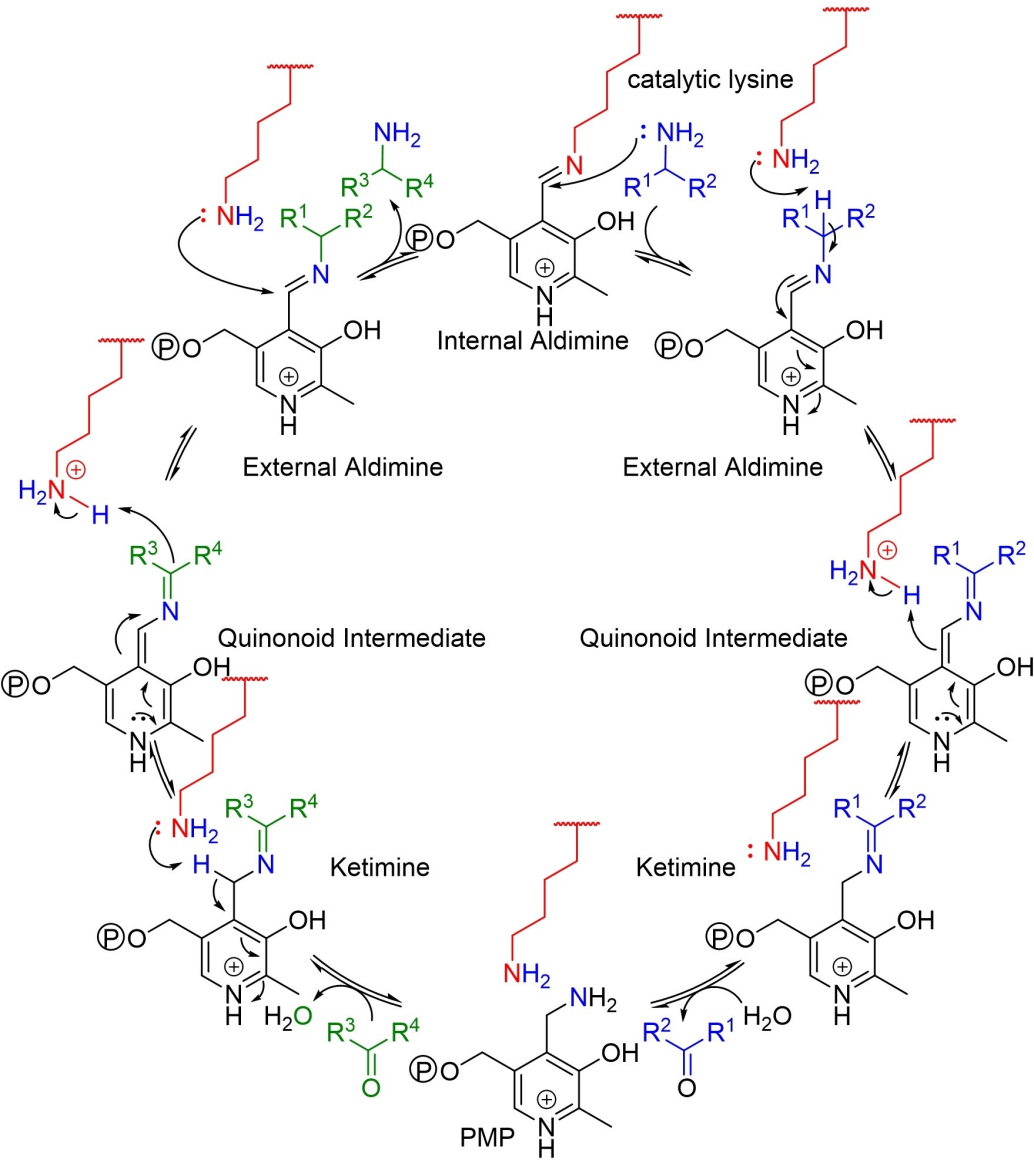
Mechanism of transamination. For clarity, the individual steps of aldimine/ketimine formation and hydrolysis as well as transimination are not shown. The mechanism is symmetric – referred to as a “ping‐pong” bi‐bi or shuttle mechanism – and fully reversible. Note: the ketimine intermediates are a second aldimine if one of the R‐groups is a hydrogen. Catalytic lysine: red; amine donor: blue; ketone acceptor: green.

The chemistries of those co‐factors could be investigated in the absence as well as in the presence of their enzymes, and from this, mechanistic details could be inferred. In addition, structural analogues could be synthesized and their reactivities compared. For example, careful isotope labelling studies in the early 1950s revealed that one hydride of the pyridine ring of NAD(P)H was transferred during reduction/oxidation in a stereospecific manner, giving additional detail to Otto Warburg's mechanism (Figure 6).^[41]^ In 1957, Breslow showed by NMR that an anion in position 2 of a thiazolium ring could exist, revealing the reactive center of thiamine (Figure 6).^[42]^ The observation that pyridoxal, the co‐factor of transaminases, as well as structural analogues with electron withdrawing groups on the aromatic ring, can catalyze transamination in the absence of the enzyme allowed Alexander Braunstein and Esmond Snell to postulate independently a likely catalytic cycle in 1954, which later proved to be correct (Figure 7).[^33b^, ^36b^, ^43^]

**Scheme 2:**
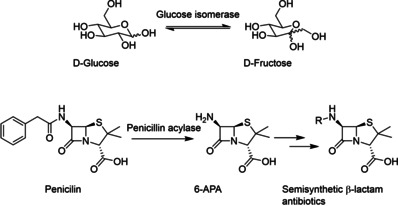
Top: Isomerization of d‐glucose to d‐fructose, catalyzed by an immobilized glucose isomerase as used in the production of HFCS. Bottom: Hydrolysis of Penicillin to give 6‐APA, which can then be acylated to give several semi‐synthetic antibiotics.

However, these advances in the knowledge of how enzymes work had no immediate impact on industrial biocatalysis, which was largely limited by the low quantities most enzymes could be obtained in. Major developments at the time include the application of glucose isomerase for the production of high fructose corn syrup (HFCS) and the development of a penicillin acylase process for the production of 6‐aminopenicillanic acid (6‐APA, at the time obtained from chemical cleavage of penicillin), a building block for semi‐synthetic antibiotics such as ampicillin and amoxycillin (Scheme 2).^[44]^ Key for the success of both applications was the discovery of the possibility to immobilize proteins with retention of their function as discovered in the 1950s.^[45]^ This allowed the enzymes to be recycled and used in a continuous fashion, reducing cost by reducing the quantity of enzyme that has to be isolated. The HFCS processes became wide‐spread in the 1970s.^[46]^ However, the production of 6‐APA via chemical hydrolysis predominated until the early 1990s, at least partially due to the difficulty of obtaining sufficient quantities of penicillin acylase before then.^[47]^ A notable exception is Bayer, who used an immobilized penicillin acylase since 1972 as a closely guarded secret, employing *E. coli* strains that achieved a penicillin acylase content of ca. 20 %.^[48]^ Processes to synthesize amino acids using immobilized enzymes (as well as whole cells) were being commercialized in Japan from 1973.^[49]^

## 4 The DNA revolution

In order for enzymes to enjoy more widespread use, their production had to be ramped up dramatically. In addition, to apply the insights into enzyme mechanisms described above, enzyme active sites had to be tweaked somehow. The key to both these problems was the understanding of how DNA encodes proteins, as well as the development of efficient ways of manipulating DNA. Of course, in parallel to the research into proteins described above, research into DNA was also ongoing. While DNA was originally viewed as less important than proteins (due to its simple make‐up of four building blocks), this view quickly changed with the discovery that it was the carrier of hereditary information by Avery in 1944.^[50]^ Of course, the correct structure of DNA was postulated in 1953 by Watson and Crick^[51]^ (Nobel Prize in Physiology or Medicine in 1962) from X‐ray diffraction data by Rosalind Franklin.

**Figure 8:**
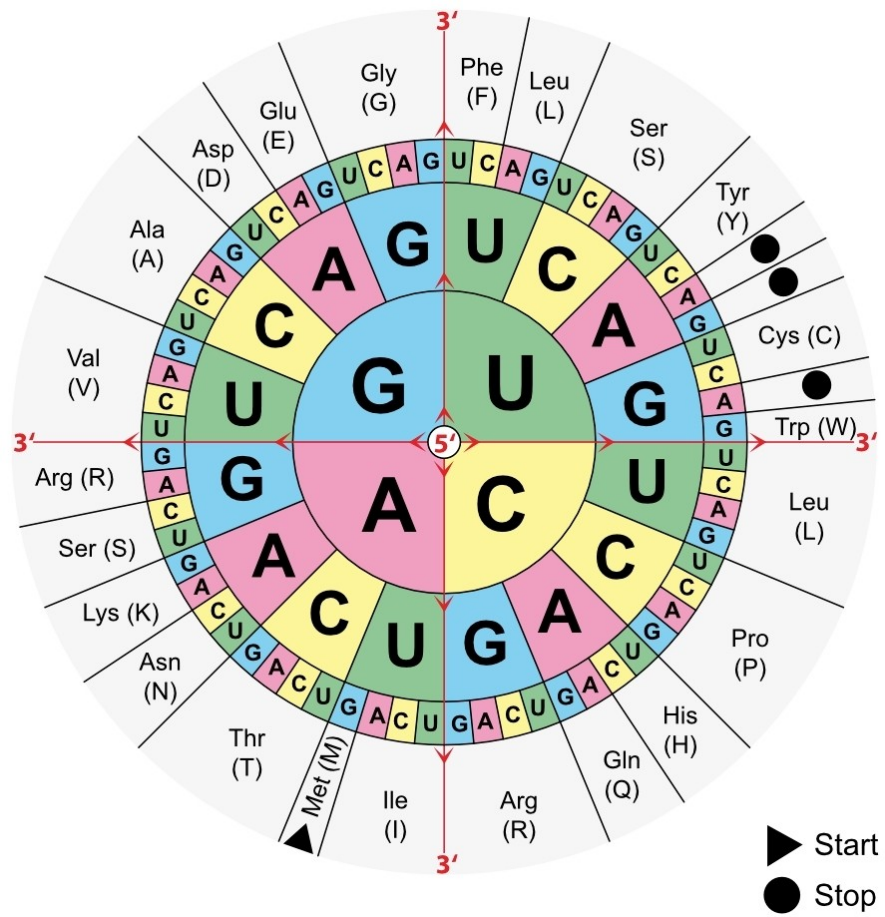
The genetic code. Codons consisting of three bases (triplets) correspond to different amino acids. Each amino acid may be spelled by multiple codons (the code is degenerate). The chart is read from the inside outwards (following the red arrows), e. g. “AUG” corresponds to the start codon, methionine, corresponding to the beginning of a protein. The genetic code is universal, i. e. identical across all organisms with only a few exceptions. https://commons.wikimedia.org/wiki/File:Aminoacids_table.svg, public domain.

This quickly led to a postulation of how genetic information is encoded in DNA: the hypothesis that DNA encodes amino acid sequences^[26e]^ and that the amino acid sequence alone determines the structure of proteins, as demonstrated by Anfinsen in 1961^[52]^ (Nobel Prize in Chemistry in 1972). The process of transcription of DNA into mRNA and the translation of mRNA into protein itself was broadly solved within a decade of the discovery of the structure of DNA; transcription as a concept was proposed by François Jacob and Jacques Monod in 1961^[53]^ (Nobel Prize in Physiology or Medicine in 1965). In the same year, mRNA was discovered,^[54]^ the triplet code was established,^[55]^ and the first codon (UUU) was solved by Nirenberg.^[56]^ In competition with several other groups,^[57]^ amongst them Gobind Khorana developing a sequence specific chemical synthesis of polynucleotides, the genetic code (Figure 8) was fully solved by 1966 and its universality established by 1967^[58]^ (Nirenberg, Khorana and Robert W. Holley (for the isolation of tRNA) received the Nobel Prize in Physiology or Medicine in 1968).

**Figure 9:**
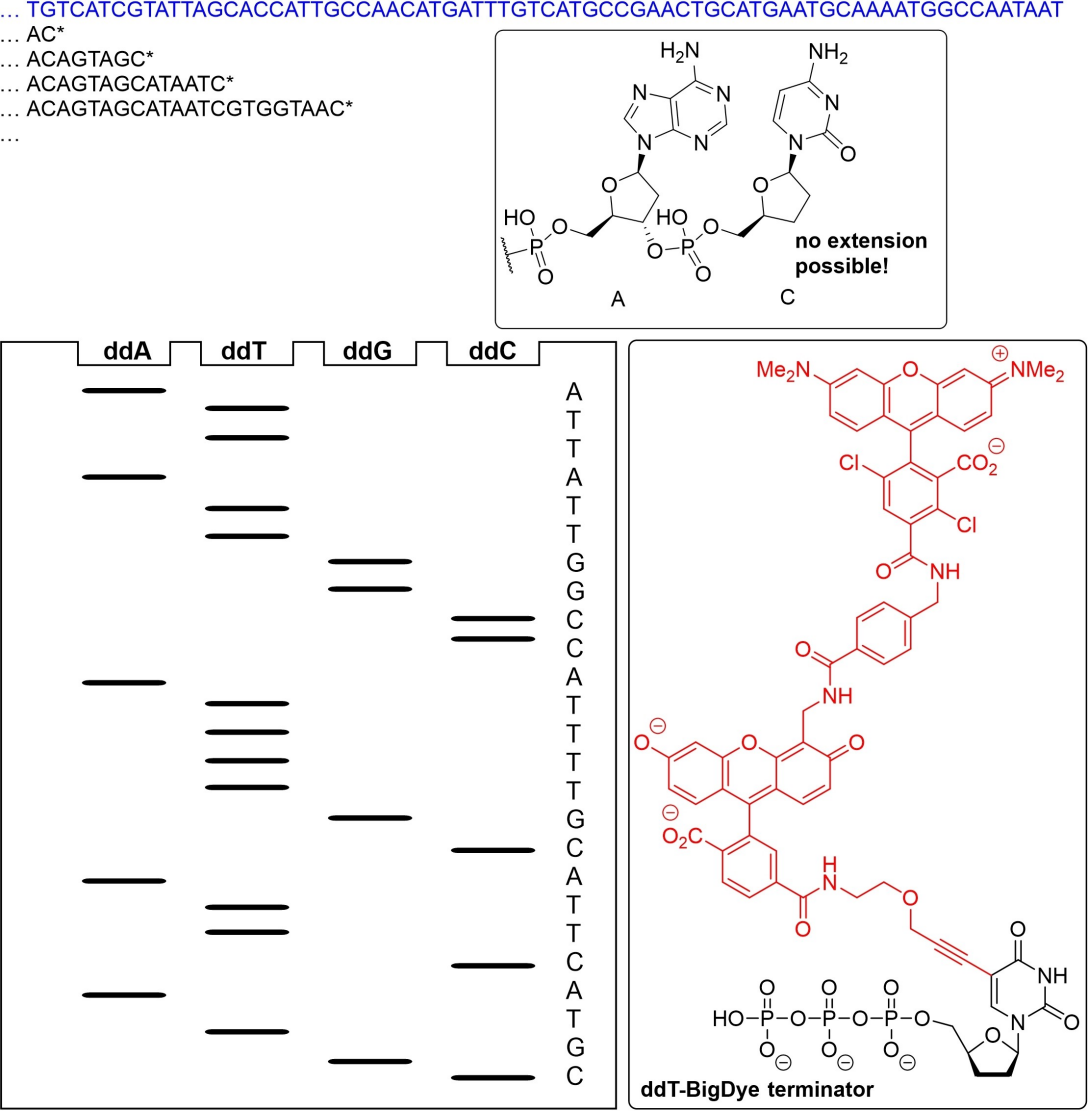
Principle of Sanger sequencing: A DNA strand (blue) is copied by DNA polymerase. If a small quantity of dideoxynucleotides (ddNTP) is offered in addition to deoxynucleotides (for example ddCTP), chains will terminated whenever a ddCTP is incorporated instead of a dCTP, as the 3′‐OH needed for chain‐extension is missing. If this experiment is repeated for all four nucleotides, and the products are separated by size, the sequence of the DNA template can be inferred. Modern Sanger sequencing includes all four ddNTPs in a single sequencing reaction, and distinguishes incorporation of the different bases at the termination site via fluorescent labels, such as the label (red) for the ddT – BigDye terminator shown.^[60]^

Understanding the meaning of the genetic code of course is of limited use, unless one can also read the DNA sequence. Frederick Sanger developed an ingenious hi‐jacking of normal DNA replication in 1977 (Figure 9):^[59]^ by supplying a small quantity of nucleotides that could not be further extended (because they missed the 3′‐OH), strands of DNA truncated after every A (or C, G, or T; depending on which was supplied as the dideoxynucleotide) were produced, which could be separated by size using gel electrophoresis. Repeating this experiment for all four nucleotides, the sequence of bases in the template could be deduced. This is earned Frederick Sanger his second Noble Prize in Chemistry in 1980. While initially DNA was visualized using radioactive labels, this was quickly replaced by using fluorescently labelled dideoxynucleotides, allowing for all four bases to be present in the same reaction mixture, increasing throughput. Using capillary electrophoresis, automated sequencing became possible.^[60]^ Next‐generation sequencing, introduced in the mid‐2000s, involves the massive parallelized sequencing of many smaller DNA segments that are then assembled *in‐silico*.^[61]^ This has significantly reduced the cost and time of genome sequencing, and thus resulted in a dramatic increase of available genomes, with over 55000 genomes deposited in the NCBI database, as of August 2020.^[62]^

**Figure 10:**
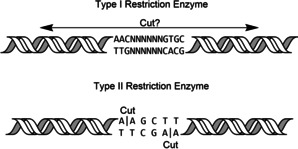
Type I restriction enzymes cut at a non‐defined remote location from the recognition site (example of EcoKI). Type II restriction enzymes cut at a well‐defined position within (or close to) the recognition sequence, often in a staggered way, producing cohesive ends (example of HindIII).

At the same time and leading on from Avery's experiment, the transmission of genetic information in bacteria was being investigated. In 1952, Joshua Lederberg coined the term “plasmid” to describe such transmissible DNA and discovered the nature of its transmission (Nobel Prize in Physiology or Medicine in 1958).^[63]^ Also in 1952, Salvador Luria^[64]^ and Giuseppe Bertani^[65]^ observed that bacteriophages from one strain of *E. coli* have a decreased virulence in another, but upon growth in the second strain would show increased virulence for it and a decreased virulence for the original strain, observing the effect of restriction enzymes (so called because they restrict the growth of bacteriophage). This effect was then also observed for several other bacteria. However, it was only in the early 1960s that the nature of these enzymes as site specific endonucleases, and that host bacteria protect their own DNA through modification (methylation), was proposed by Werner Arber.^[66]^ Mathew Meselson isolated the first such restriction endonuclease in 1968 (EcoK1).^[67]^ These early restriction enzymes recognized a specific sequence but did not cut in a specific location, and are now known as type I restriction enzymes. Type II restriction enzymes, cutting DNA in specific locations (Figure 10), were discovered by Hamilton Smith in 1970 (HindII and HindIII).^[68]^ In conjunction with gel electrophoresis, this allowed the digestion of DNA into fragments of defined size which could then be separated, as shown by Daniel Nathans in 1971.^[69]^ Arber, Smith, and Nathans won the Nobel Prize in Physiology or Medicine in 1978.

**Figure 11:**
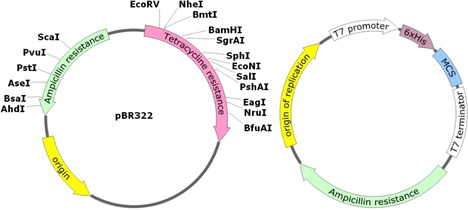
Left: Map of pBR322, showing the unique restriction sites inside both antibiotic resistance genes. Right: Generic map of a typical (empty) expression vector, having an origin of replication (for replication *in vivo)*, a selective marker (Ampicillin resistance in this instance), and the T7 promoter and terminator flanking a His‐tag (to allow purification of the insert) and the multiple cloning site, which contains a large number of unique restriction sites (not shown) for easy cloning.

As many type II restriction enzymes produce palindromic single stranded overhangs, it was then realized that DNA from different sources could be stitched together if cut with the same restriction enzyme, through the action of DNA ligase. The first such “recombinant” DNA was reported by Paul Berg in 1972 (Nobel Prize in Chemistry in 1980).^[70]^ It thus became possible to introduce any piece of DNA from any organism into (for example) *E. coli*.^[71]^ Thus, plasmids for convenient introduction of such recombinant DNA were being developed in the 1970s.^[70c]^ On of the most famous of these plasmids is pBR322, developed by Bolivar and Rodriguez (BR) in 1977.[^70c^, ^72^] This plasmid made use of two antibiotic resistance genes and several unique restriction sites within them to allow for the selection of colonies that had a) up‐taken the plasmid and b) up‐taken a plasmid containing an insert (Figure 11). The propagation of recombinant DNA in a new host is referred to as cloning. The pUC series of plasmids (UC for University of California), derived from pBR322, allowed for colorimetric detection of inserts.^[73]^ Finally, the pET series of vectors, also derived from pBR322, was created in the late 1980s and included a T7 promotor, allowing for the selective expression of the DNA insert (ET for Expression by T7 RNA Polymerase).[^74a^, ^74b^] Strains of *E. coli* were generated containing the gene for the T7 polymerase, under the control of a modified *lac* promoter (lacUV5), as a lysogen of the DE3 phage.[^74b^, ^74c^, ^74d^, ^74e^] Alternative promotor systems were also developed, such as the aforementioned *lac* promoter, as well as the *trc* promoter, *p*L promoter, and *tet*A promoter, and more, each with their own advantages and draw‐backs.^[75]^

**Scheme 3:**
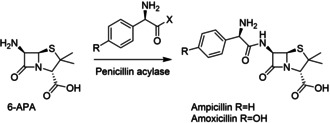
Application of penicillin acylase for the synthesis of amoxicillin and ampicillin from 6‐APA. X=NH_2_ or OMe.

These developments in molecular biology revolutionized enzymology and biocatalysis. For the first time, the DNA sequence of an enzyme of interest could be determined and cloned, and the enzyme could be over‐expressed in *E. coli* (or another suitable organism) and thus be obtained in sufficient quantities to be studied and used in industrial applications. The first recombinant protein produced was insulin in 1978, and the commercial production of human insulin started in 1982.^[76]^ Prior to that, insulin had to be isolated from pigs or cows and often had limited and inconsistent efficacy, as well as inconsistent supply.^[77]^ DNA recombinant technology also allowed penicillin acylase to be obtained in sufficient quantities and enabled its widespread application toward the synthesis of 6‐APA, as mentioned above.[^45e^, ^48^] Indeed, penicillin acylase was one of the first enzymes expressed recombinantly, in 1979, only one year after insulin.[^45d^, ^48^] The availability of this enzyme also enabled the development of its application in the reverse direction, catalyzing the amide bond formed between 6‐APA and the side chains found in semi‐synthetic antibiotics such as amoxicillin and ampicillin (Scheme 3, also see below for a discussion of the role of immobilization).[^44^, ^47^, ^78^] Around the same time, recombinant chymosin (which selectively hydrolyzes casein between residues F105 and M106, resulting in the curdling of milk) started replacing natural rennet, obtained from calf stomachs, in cheese‐making.^[79]^ This provided a cheaper, more stable supply for cheesemaking as well as more consistent results due to a higher purity. By 2006, up to 80 % of all rennet was recombinant chymosin and cheese production in the US had increased over two‐fold.^[80]^

## 5 Directed evolution and the beginnings of modern biocatalysis

**Figure 12:**
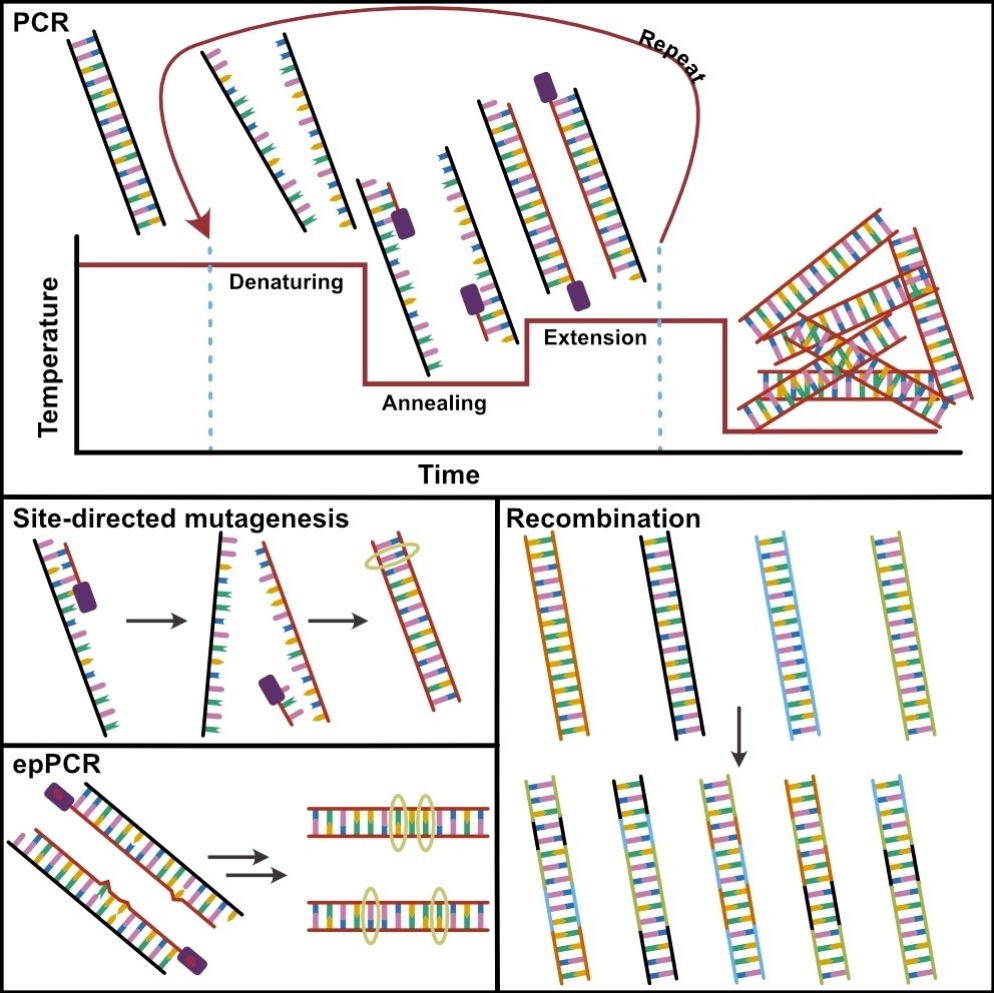
The principles of PCR: DNA is denatured at high temperature, primers supplied in the reaction mixture are annealed, and the template is copied. Repeated cycles exponentially amplify the target sequence. Site‐directed mutagenesis: a mutation is incorporated in the primer; the amplified product now contains the changed base‐pair. epPCR: a polymerase that occasionally incorporates incorrect nucleotides is used. The product now contains a set of different sequences that differ from the parent in a few positions. Recombination: several sequences are shuffled to produce a diverse set of new sequences from the parents.

As great as natural enzymes are at carrying out their function, they often present drawbacks which make them unsuitable for industrial applications such as their lack of stability, (co‐)solvent‐tolerance, or a very limited substrate scope. While immobilization can address the stability problems (see below),^[81]^ it quickly became desirable to be able to change the properties of the enzymes themselves. Of course, to some extend this had already been done routinely, through strain optimization. Whole organisms were subjected to mutation‐inducing conditions, such as radiation or chemical agents, and the resulting strains were screened for favorable phenotypes.^[82]^ Through this method, strains producing larger quantities of desirable products, either specific enzymes (such as in the case of penicillin acylase at Bayer mentioned above) or chemicals could be obtained, and entirely new pathways could be introduced.^[83]^ However, these approaches were slow, unlikely to directly change the properties of any specific enzyme, and could only really be applied to organisms with sufficiently short replication‐cycles. However, with the availability of recombinant DNA, as well as the understanding of enzymes and their mechanisms as outlined above, introduction of specific mutations into a target enzyme was now within reach, irrespective of the organism it originated from. Indeed, a general method for site‐directed mutagenesis was being reported by Michael Smith in 1978 (Nobel Prize in Chemistry in 1993), the same year as the cloning of insulin was achieved. By designing DNA primers, harboring the desired mutations, complimentary to the target sequence to be mutated, and extending with DNA polymerase using the target sequence as a template, copies containing the mutation could be made (Figure 12).^[84]^ Of course, efficient syntheses of specific DNA sequences such as those developed by Khorana,^[85]^ Gillam,^[86]^ and Caruthers^[87]^ were instrumental for this.^[88]^

Using this approach, the role of catalytic residues could be directly investigated. For example changing the cysteine in the catalytic triad found in tyrosyl tRNA synthase to a serine (as found in esterases mentioned above) greatly reduces the efficiency of the enzyme.^[89]^ Indeed, the role of several residues in several enzymes could now be quantified, confirming and in some cases revising mechanisms that had been postulated based on crystallography.^[90a]^ However, using this technique to introduce desirable properties into enzymes was quickly met with the realization that the effect of mutations was often unpredictable, and rational engineering of enzymes was often not successful. However, a shift was quickly made toward a more random approach such as the use of site‐saturation mutagenesis, where targeted residues were changed to all possible amino acids rather than a specific one. Through this, some progress was made such as the introduction of a stabilizing mutation into subtilisin, a protease with application in laundry detergents,[^81^, ^90^] or enhanced thermostability of glucose isomerase.^[91]^

With the development of the polymerase chain reaction (PCR, Figure 12) in the 1980s by Kary Mullis (Nobel Prize in Chemistry in 1993), it became possible to produce large numbers of copies of DNA sequences from a single template.^[92]^ By modulating the fidelity of the polymerase, random mutations could be introduced into the amplified product (error‐prone or epPCR, Figure 12). In the early 1990s, Frances Arnold used this technique to create large libraries of mutants to which she then applied evolutionary pressure. In her own words,^[93]^ she

“rejected microbial growth or survival selections favored by microbiologists and geneticists. Thus we turned to good old‐fashioned analytical chemistry to develop reproducible, reliable screens that reported what mattered to us.”

In doing so she managed to produce a variant of subtilisin E that could tolerate high concentrations of DMF, introducing a total of 10 mutations.^[94]^ Thus, the field of directed evolution was born. In 1994, Pim Stemmer introduced the concept of DNA shuffling (Figure 12), mimicking DNA recombination which occurs in organisms as a way to increase genetic diversity, and applying it to recombinant DNA *in vitro*. Without being restricted to genes from a single species, very diverse proteins could be mixed together to create new sequences very distant from natural ones.^[95]^ This technique proved very powerful on its own, but especially when combined with epPCR, allowing the combination of mutations from several mutants without the need for any understanding of how the different mutations would interact with each other.^[96]^ Frances Arnold received the Nobel Prize in Chemistry in 2018 for the directed evolution of enzymes. In addition to co‐solvent tolerance, directed evolution was quickly used to create enzymes with improved thermostability,[^97a^, ^97b^, ^97c^, ^97d^] pH stability,^[97c]^ as well as enhanced activity at low temperatures,[^97c^, ^97e^] activity toward unnatural substrates,[^96^, ^98^] modified enantioselectivity,^[99]^ or combinations of the above. Thus, it quickly established itself as a powerful tool in protein engineering across structurally and functionally diverse classes of enzymes. Using random mutagenesis methods it was quickly realized that beneficial mutations were often found in unexpected parts of the enzymes, explaining why early rational attempts struggled at accomplishing these modifications.[^81^, ^93^, ^100^]

**Figure 13:**
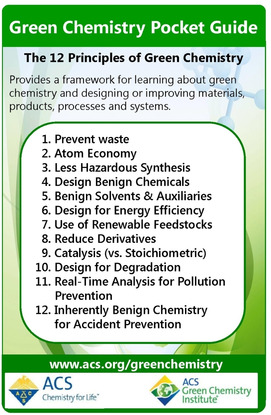
The 12 principles of green chemistry, reproduced with permission from ACS Green Chemistry Institute® (https://www.acs.org/content/acs/en/greenchemistry/principles/12‐principles‐of‐green‐chemistry.html). Copyright 2020 American Chemical Society.

The sudden availability of biocatalysts with properties suitable for industrial applications, as well as the ability to create those properties at will, made them very attractive for use in synthetic applications that so far had been considered out of reach.^[101]^ Of course, catalysis itself had become a major field of interest in synthetic chemistry in the second half of the 20^th^ century, as the environmental impact of traditional (stoichiometric) chemistry was gaining attention.^[102]^ This gained more traction with the conceptual development of green chemistry in the 1990, in parallel to the advances made in biocatalysis outlined above.^[103]^ Thus, it is not surprising that biocatalysis formed a key strategy of accomplishing the goals of green chemistry from the start.^[102b]^ Indeed, it promises to address many of the “12 principles of green chemistry” (Figure 13), in particular with regard to hazardous reagents and waste, energy requirements, number of steps, and their inherently renewable and biodegradable nature.^[104]^ Indeed, the number of biocatalytic processes in industry started increasing rapidly and continues to do so to this day: there were around 60 processes in 1990, 134 processes in 2002, and several hundred by 2019.^[105]^

**Scheme 4:**
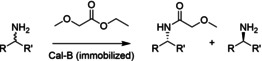
The lipase‐catalyzed BASF process for the kinetic resolution of amines. Enantioselectivity is often essentially perfect and conversions quantitative, the amide and amine can be separated by distillation, the amide is readily hydrolyzed (giving access to both enantiomers), and the undesired enantiomer can be racemized and recycled. The process can also be run neat (e. g. in the case of 1‐methoxy‐2‐aminopropane).^[108b]^ Other esters than the ethyl may be used; however, the methoxy‐group is critical for an efficient reaction.

Perhaps one of the most successful examples developed at the time was the use of lipases, in particularly CalB from *Candida antarctica*, in organic solvents, allowing ester and amide formation without competing hydrolysis, which is frequently employed in (dynamic) kinetic resolutions of chiral alcohols and amines. The latter was developed at BASF^[106]^ and is often referred to as the “BASF process” or “ChiPros technology” (Scheme 4).^[107]^ By 2004, multiple BASF plants produced chiral amines on a >1000 ton scale per year, and this process is still in use today. The reactions can be carried out without solvent, are often nearly quantitative, both amine and amide are readily isolated, and the undesired product can be recycled, making this process highly efficient.^[108]^

## 6 “Smart” libraries and applications of enzyme engineering

While the BASF process uses a wild‐type enzyme, stabilized through immobilization, a strong interest in enzyme engineering has developed in industry. One major barrier to directed evolution is screening. In Frances Arnold's original paper,^[94a]^ the enzyme was secreted from colonies of bacteria and digestion of the substrate in plates could be easily observed by a decrease in turbidity, thus allowing the screening of a large number of variants relatively easily. However, in general screening is not straightforward and usually it is the bottleneck. This is tackled on two fronts: development of faster high‐throughput screens, as well as reduction of the size of libraries by increasing the proportion of hits. While the former is often highly specific to the (class of) enzymes being evolved, more general concepts exist for the latter.^[109]^ For example, the structure of the enzyme may be used to assess which recombinations are more likely not to disrupt the overall fold of the enzyme, in a process called SCHEMA,^[110]^ which can then be used to reduce the size of combinatorial libraries.

Advances in the understanding of protein structures as well as dynamics have increasingly allowed target residues to be identified with more reliability than was previously possible, reducing the need for random mutagenesis across the whole gene although it remains a valuable tool.^[111]^ This is accomplished using increasingly sophisticated bioinformatic tools, to allow for the docking of substrates into active sites, molecular dynamic simulations, and protein structure modeling, which can aid to predict the likely effect of potential mutations. One very powerful tool that has emerged is Rosetta, which also has been applied for the complete *de‐novo* design of proteins.^[112]^ The availability of increasing numbers of structures of diverse enzymes within a given family, and the even larger availability of sequences (due to next generation high‐throughput sequencing technologies) allow points of natural variation to be identified which may then be targeted. The flexibility of residues as determined by X‐ray crystallography may also help identify target regions.^[113]^ Alternatively, random mutagenesis might be used to identify hotspots which are then further investigated by more targeted mutagenesis.^[114]^ In addition, the amino acids found in nature for a given position can inform which substitutions to include in a given library.^[115]^

Several residues may be targeted together, to increase the chance of detecting synergistic effects of mutations. One such approach is combinatorial active site testing (CASTing), developed by Manfred Reetz,^[116]^ whereby multiple residues lining the active site are saturated at the same time, allowing for synergistic effects between mutations to emerge. This has been particularly successful in changing enantioselectivities and substrate scopes of enzymes.[^107^, ^117^] Amine dehydrogenases (AmDH) were created from amino acid dehydrogenases in this way.^[118]^ Several sites of interest (each potentially consisting of multiple residues) may be targeted sequentially, in a process call Iterative Saturation Mutagenesis (ISM), also developed by Reetz.^[119]^

Statistical tools and machine learning are also a powerful way to increase the efficiency of directed evolution,^[120]^ such as the use of protein sequence activity relationships (ProSAR).^[121]^ In an initial library, mutations are classified as beneficial, neutral, or detrimental and can inform which mutations to incorporate into subsequent libraries, as opposed to taking the best overall variant and generating a new library. This strategy was successfully applied by Codexis in the engineering of a halohydrin dehalogenase (HHDH) for the synthesis of (*R*)‐4‐cyano‐3‐hydroxybutyrate, a key intermediate for the synthesis of atorvastatin, a cholesterol‐lowering drug.^[122]^ Overall, the volumetric productivity was improved 4000‐fold over 18 rounds of evolution and 35 mutations were introduced, meeting the process requirements for the enzyme. The authors note that half of the mutations introduced in the final variant were initially not present in the best variant when selected and would have been missed in a hit‐based approach. While this approach can reduce screening efforts, it requires a larger sequencing effort. However, this has become increasingly possible as the cost of DNA sequencing has steadily declined.^[81]^

**Figure 14:**
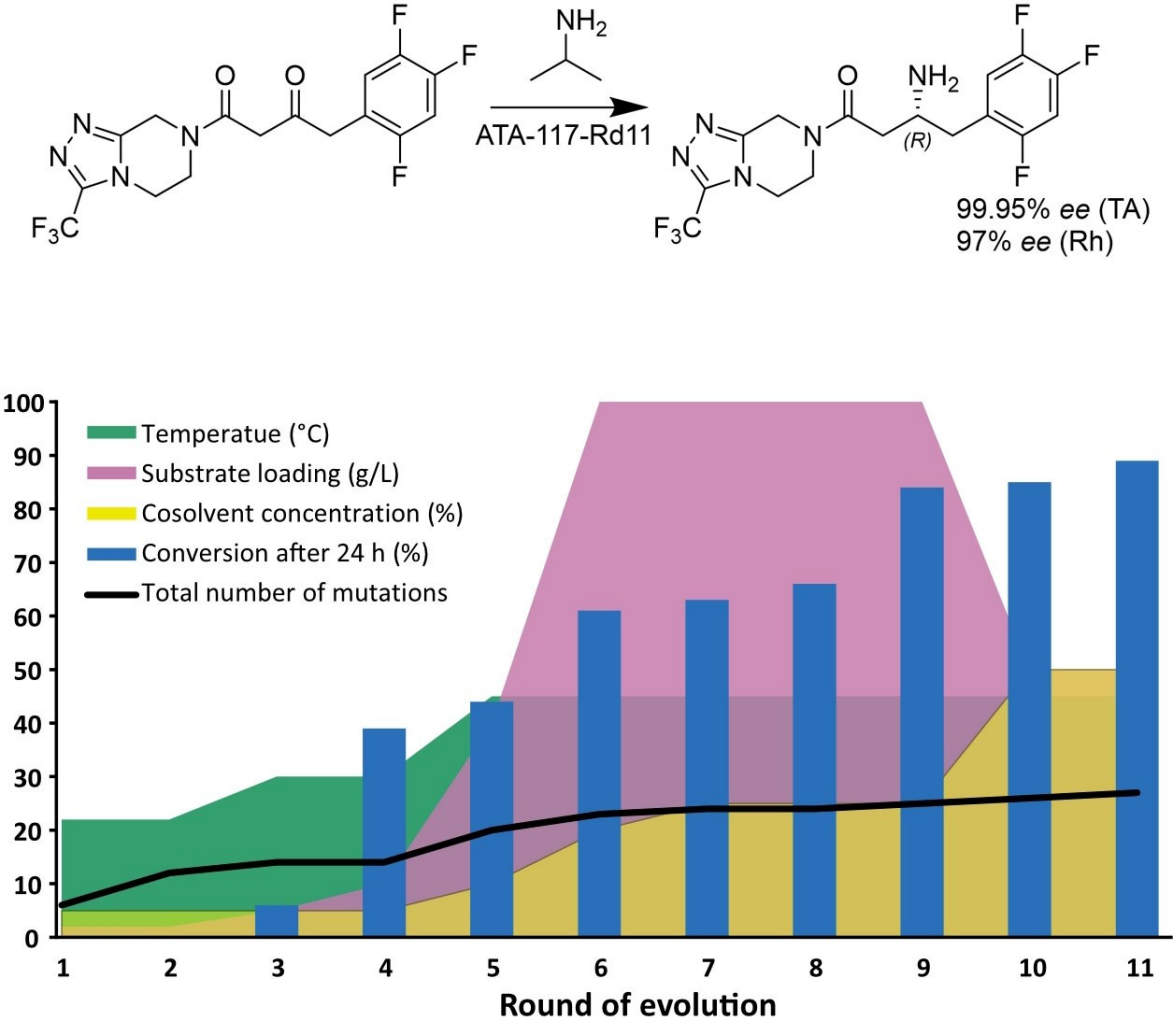
Evolution of ATA‐117 to produce sitagliptin,^[123]^ compared to the chemocatalytic route using a rhodium catalyst. Over 11 rounds of evolution, the conditions of the screening (substrate loading, temperature, cosolvent concentration (Rd 3–6 MeOH, otherwise DMSO) were gradually increased to the process level. Overlaid is the steady increase in conversion under process conditions, as well as the increase in the total number of mutations (note, several mutations changed throughout the process).

Codexis and Merck combined several of these approaches to engineer a transaminase for the synthesis of sitagliptin. Starting from an enzyme with no activity toward the substrate and minimal activity toward a truncated analogue, 11 rounds of engineering (Figure 14) led to a catalyst that outcompeted the alternative rhodium‐catalyzed reductive amination process in terms of efficiency, yield, enantioselectivity, and waste formation. Overall, 27 mutations were introduced using a combination of site‐saturation mutagenesis, combinatorial libraries (including diversity from homologous sequences), proSAR, and epPCR, screening a total of 36480 variants.^[123]^ Highlighting that, even with the use of tools to maximize the efficiency of evolution, a huge screening effort may still be required for significant catalyst improvements.

**Figure 15:**
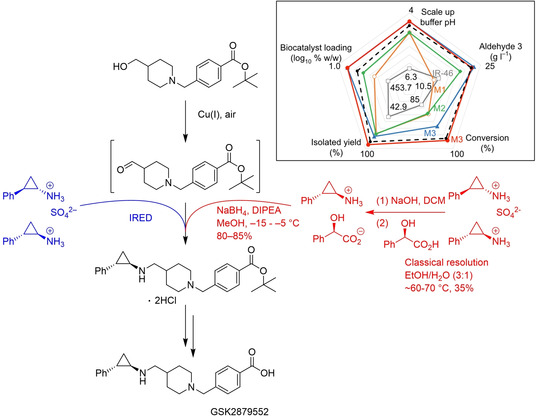
Engineering of an IRED for the synthesis of GSK2879552, and the alternative chemical route. Insert: improvement of the catalyst over 3 rounds of evolution; acceptable operating space (black dotted line), wild type IR‐46 (grey), M1 (orange), M2 (green), M3 at small scale (blue) and process scale (red). Adapted from Schober *et al*.^[124]^

In another more recent example, GSK evolved an imine reductase (IRED) to meet the process requirements for the synthesis of the LSD1 inhibitor GSK2879552, currently in clinical trials.^[124]^ Screening of their in‐house panel (of at least 85 IREDS)^[125]^ revealed a suitable candidate for mutagenesis. Given the scarcity of structural data on IREDs and that their highly dynamic mechanism is not fully understood, an initial round of site‐saturation mutagenesis was carried out on 256 out of 296 positions. Beneficial mutations from that round were then used to generate combinatorial libraries, which were then analyzed using the proprietary CodeEvolver software from Codexis. Statistical analysis was performed to identify pairwise interactions of beneficial mutations which were then included in another combinatorial library in a final third round of evolution, yielding an enzyme with 13 mutations that met or exceeded the process requirements, resulting in improved sustainability metrics over the previous route (Figure 15). The enzyme was then used to synthesize 1.4 kg of GSK2879552 for use in additional rounds of clinical trials.

**Figure 16:**
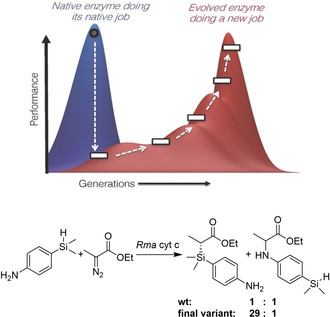
Top: Exploiting enzyme promiscuity to evolve new catalytic activity. Enzymes often exhibit promiscuous activity toward non‐native substrates or reactions. By applying directed evolution, a “specialist” enzyme might be transformed into another specialist enzyme for the new activity, at the cost of diminishing its original function. Such a transformation proceeds through a “low‐fitness valley” where the enzyme is not very good at either the new or the original function. Figure reproduced from ref.^[93]^ Bottom: This concept was applied to evolve a cytochrome c from *Rhodothermus marinus* (without any native catalytic function) to catalyze Si−H carbene insertions.^[126]^ The final variant was 33x more active than the parent and became more specialized for Si−H insertion over N−H insertion chemistry, both promiscuous activities of the wt.

Increasingly, directed evolution has also been applied to create enzymes carrying out reactions not observed in nature, by exploiting the promiscuous nature of enzymes (Figure 16). Frances Arnold's group reported on the evolution of a cytochrome c (cyt c),^[126]^ a protein without any catalytic role in nature, to form carbon‐silicon bonds, a reaction not observed in nature. After screening several P450 enzymes, myoglobins, and cyt c variants, they identified a cyt c from *Rhodothermus marinus* with low levels of catalytic activity for this reaction. Iterative site‐saturation mutagenesis of just three key residues – an iron coordinating methionine, and two additional residues close to the heme group – resulted in a catalyst with a total turnover number (TTN) of >1500, a>33‐fold improvement over the wild‐type (wt) and a >375‐fold improvement over free heme, outperforming the best chemical catalysts for this reaction. In addition, the turnover frequency (TOF) was increased 7‐fold, the reaction proceeded with nearly perfect enantioselectivity, and was chemoselective for carbene insertion into silanes over alcohols and amines (Figure 16).

Clearly, in addition to the generation of efficient libraries, the choice of template was key in both examples above. Indeed, modern genomics has created huge databases of genes and it has become incredibly easy to identify new sequences that likely have a given catalytic function. The dramatic reduction in cost of synthetic genes has made it possible to create panels of these sequences, in a way producing a “smart” library of sequences already pre‐selected by natural evolution. The likely function of a DNA sequence may be determined from sequence similarity to other proteins of known function (using search algorithms such as BLAST),^[127]^ as well as the identification of motifs.^[128]^ In addition, structure based searches of crystal structures of unknown function may yield new biocatalyst.^[129]^

## 7 Enzyme immobilization and flow chemistry

**Figure 17:**
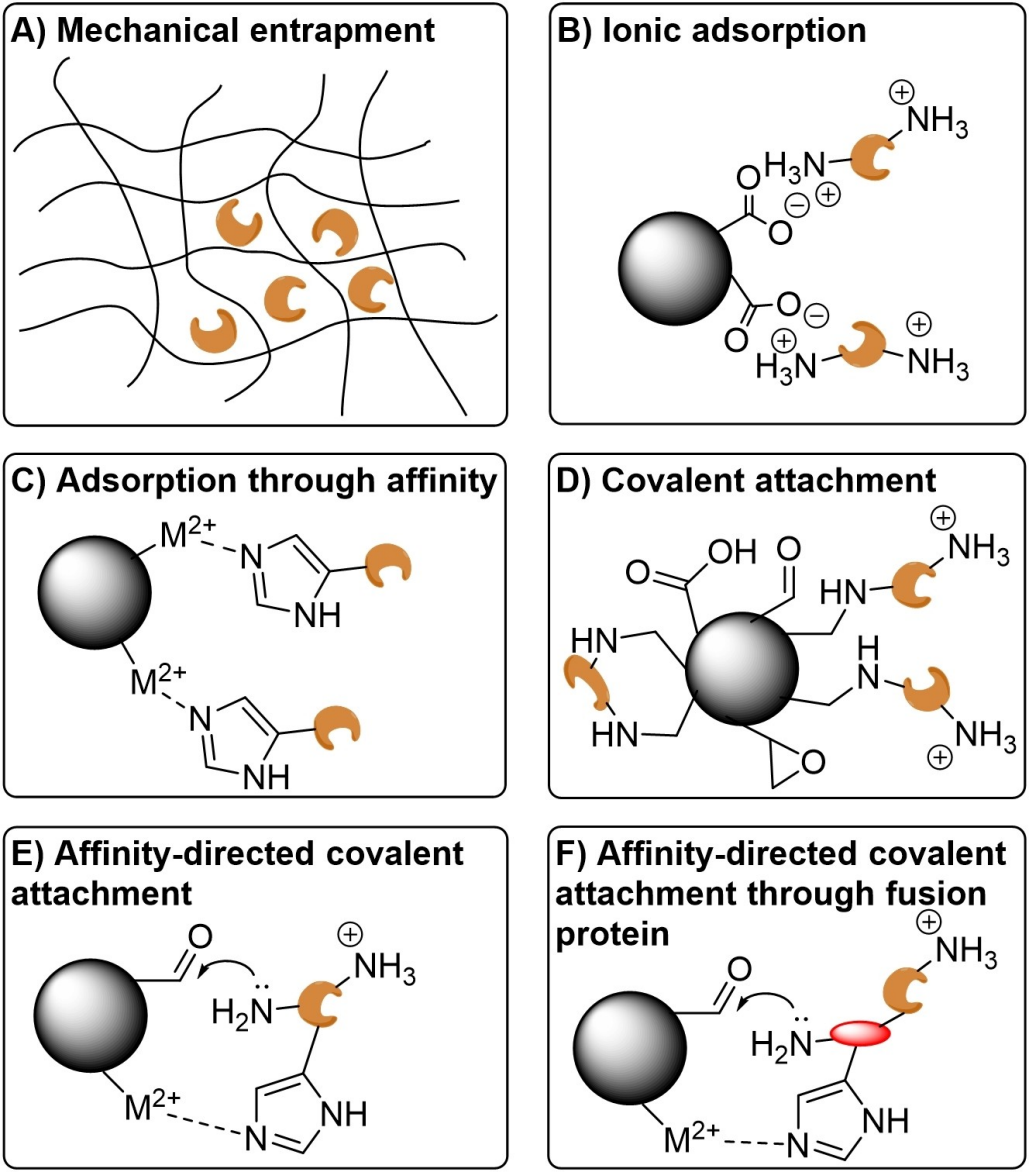
Examples of enzyme immobilization strategies. A) Mechanical entrapment restricts the diffusion of the enzyme. B) Adsorption through ionic interactions, offering little control over the orientation of the enzyme. C) Adsorption though affinity, in this case His‐tag‐metal coordination, allowing control of over the orientation of the enzyme through the tag placement. D) Covalent attachment, offering little control over the orientation of the enzyme. Multipoint attachment can lead to irreversible deformation of the enzyme shape. Common functional groups for covalent attachment are carboxylic acids, aldehydes, and epoxides – using amide formation, reductive amination, and ring opening, respectively. E) Affinity‐directed covalent immobilization orients the enzyme prior to covalent attachment. F) By fusing a (small) protein to the enzyme, covalent immobilization and any shape disruption can be localized to that fusion protein, reducing the effect on the enzyme. However, such an enzyme is more exposed to the environment and stability benefits from immobilization may be diminished. Not shown are covalent crosslinking of enzymes (e. g. using a dialdehyde), and the inherent different properties of supports, with respect to e. g. their size, pore‐size, hydrophilicity/hydrophobicity, etc.

Protein immobilization, which, as already outlined above, is a key strategy for enzyme stabilization and reusability, has also become more advanced and a whole plethora of different strategies and supports are available (Figure 17). Those are needed in part because protein immobilization can be highly unpredictable, and because of application‐dependent requirements on the immobilized catalyst (such as particle size, swelling, hydrophilicity/hydrophobicity, etc.). Enzyme immobilization may be mechanical or physiochemical, the latter can be further divided into covalent or noncovalent/adsorption immobilization. Immobilized proteins may also be used in continuous (i. e. flow chemistry) processes, which is particularly attractive for its scalability, improved efficiency, and increased control.^[130]^

Mechanical immobilization relies on the entrapment of the enzyme in a matrix that restricts its movement (Figure 17A). This has the advantage that the enzyme itself is not being modified, while allowing for its environment to be fine‐tuned. However, mass transfer to and from the enzyme is often impaired.^[131]^ Furthermore, leaching of the enzyme can occur.^[132]^ Adsorption onto a solid support is another simple immobilization strategy. However, it too often suffers from leaching of the enzyme from the support. It its simplest form (hydrophobic, hydrophilic or ionic interactions between support and enzyme. Figure 17B), no control over the orientation of the enzyme is achieved and the entrance to the active site may become blocked.^[133]^ One of the most widely used biocatalysts, CalB, is immobilized in this way (Novozym 435), through hydrophobic interactions between the enzyme and an acrylic resin support. While leaching is an issue in an aqueous environment, this is suppressed in the organic solvents in which it is usually used.^[134]^

More specific adsorption is possible with the use of tags. Attached at either the N‐ or C‐terminal of the protein, they can help orient the enzyme in a favorable position. Examples include the use of a polyhistidine‐tag (His‐tag, Figure 17C), originally developed for efficient protein purification in 1988,^[135]^ which coordinates to transition metal cations, streptavidin with its remarkably high affinity for biotin, and sugar‐lectin interactions. While a His‐tag is encoded genetically, the other two examples require biotinylation or glycosylation, respectively. This also allows for enzyme purification and immobilization to be combined into a single step. While these interactions are stronger than the simple adsorption described above, low levels of leaching can still occur and pose problems for applications in flow, where any enzyme leaching from the column will be lost (as opposed to batch processes where temporarily detached enzyme remains in the vicinity of the support and can, in principle, reattach). One example are EziG beads, made of controlled porosity glass which has been modified to coordinate to metal ions.^[136a]^ They appear to be predominantly used in organic solvents,^[136]^ which appear to suppress leaching although use in an aqueous environment without leaching has also been reported (using Fe^3+^ as the cation).^[137]^

The problem of leaching can be fully avoided using covalent immobilization (Figure 17D). However, it often results in severe distortions of the enzyme and loss of activity, although this is highly dependent on the enzyme and support and often unpredictable. Leaching may also still occur for multimeric proteins if not all subunits are covalently attached. Moreover, once the enzyme has degraded the support cannot be reused whereas with adsorption the enzyme may be desorbed and replaced with fresh enzyme.^[134]^ The orientation of the enzyme may be controlled by initially adsorbing the enzyme onto the support using tags, followed by the formation of the covalent attachment (Figure 17E). The distortion of the enzyme can be alleviated using small protein tags, with the covalent attachment points being on that protein rather than the enzyme itself (Figure 17F).^[138]^ However, as the enzyme is more exposed to solvent, the stabilizing effect of immobilization is reduced. One elegant tag directed covalent immobilization is the use of the SpyTag/SpyCatcher system, a peptide and protein that spontaneously form an isopeptide bond when coming together.^[139]^

**Scheme 5:**
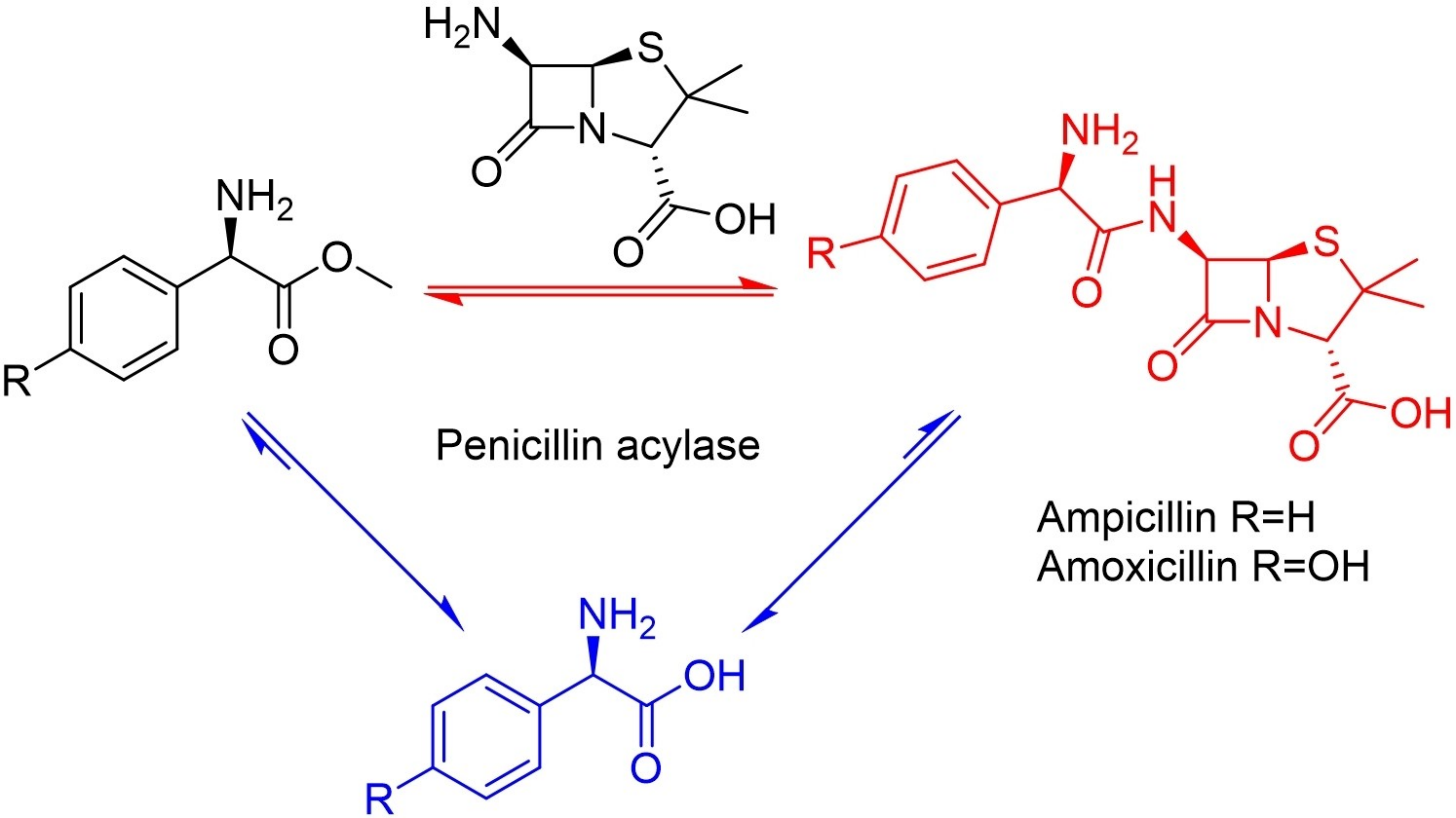
Competing acyl transfer (red) and hydrolysis reactions (blue) catalyzed by Penicillin acylase. By tuning the characteristics of the support, synthesis can be kinetically favored over the thermodynamically favored hydrolysis reaction.

Enzymes may also be covalently cross‐linked into cross‐linked enzyme aggregates (CLEAs), an immobilization without a support. However, the poorly defined properties (such as particle size) of these aggregates often render them unsuitable for flow applications. However, adsorption of these aggregates onto a support with more defined properties can alleviate this issue. Glucose isomerase, immobilized in this way, is being used for the production of HFCSs in flow on a 10 million tons per year scale, using 500 tons of the immobilized catalyst.[^45f^, ^108b^, ^140^] Regardless of the immobilization technique used, tuning the characteristics of the support with respect to e. g. their size, pore‐size, hydrophilicity/hydrophobicity, etc. is also key. For example, tuning the composition of the support reduced the synthesis/hydrolysis (S/H) ratio of covalently immobilized penicillin acylase (Scheme 5). This was a key step in rendering it suitable for the kinetically controlled synthesis of various semi‐synthetic β‐lactam antibiotics.[^44^, ^47^, ^78^]

In some cases, subunit dissociation followed by leaching can significantly reduce the operational stability of covalently immobilized biocatalysts. In those cases, coating the biocatalysts either before or after immobilization with other polymers, such as polyethylenimine (PEI) or activated dextran can help maintain the quaternary structure.^[131]^ However, as with any immobilization, excessive rigidification of the enzyme can result in a loss of catalytic efficiency if structural rearrangements necessary for catalysis are impeded. In addition, the increased complexity of more sophisticated immobilization techniques often outweighs any benefits bestowed on the catalyst. In general, the simplest catalysts that can meet the process requirements is the preferred one.

Synthetic cascades, without the need for purification of intermediates are very attractive due to the reduced amount of waste that is produced. In addition, intermediates that are too unstable to be isolated may be telescoped to the next step, offering alternative routes to classical synthesis. Flow chemistry, being inherently modular by design, is a very important platform for such cascades. Sequential reactions can be compartmentalized, avoiding incompatibility between reagents as well as allowing the conditions to be fine‐tuned for each reaction.^[130]^ Cascades involving biocatalysis may either be multiple enzymatic reactions combined in sequence, or chemo(catalytic) reactions combined with enzymatic reactions.^[141]^

**Scheme 6:**
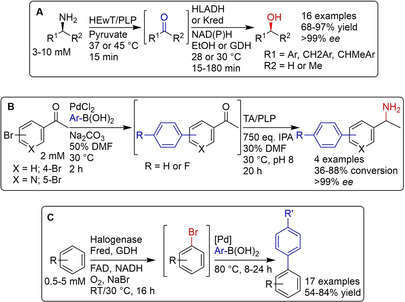
Three examples of enzymatic cascades. A) Transformation of amines into alcohols, using an immobilized transaminase and either an ADH or KRED in flow.^[142]^ B) A Suzuki cross‐coupling to produce a bi‐aryl ketone which is then aminated using a transaminase catalyst. The transaminase had to tolerate 30 % DMF carried over from the cross‐coupling, as well Pd catalyst, excess base, and unreacted boronic acid.^[143]^ C) halogenation of aromatic compounds using a halogenase, followed by a Suzuki coupling. The enzyme had to be separated, either by ultrafiltration, immobilization, or compartmentalization from the Pd catalyst.^[144]^

A nice example of the former was demonstrated by Contente and Paradisi,^[142]^ who developed a cascade in flow converting amines into alcohols, employing a transaminase and either an alcohol dehydrogenase (ADH) or ketoreductase (KRED) that had been immobilized covalently on epoxide functionalized methacrylate beads (Scheme 6A). By compartmentalizing both catalysts, reaction temperatures and times were optimized independently for each step. In addition, in‐line purification steps allowed the removal of product. Recycling of the aqueous phase containing co‐factors and buffer salts was also demonstrated, reducing the overall amount of the cofactors required (from 1 : 100 to 1 : 2000) while also eliminating the aqueous waste stream. Uwe Bornscheuer's group demonstrated a Suzuki‐Miyaura coupling in batch to produce a biaryl ketone substrate for a subsequent transaminase‐catalyzed amination in flow (Scheme 6B).^[143]^ Here, the ability of the transaminase reaction to tolerate 30 % (*v/v*) DMF as well as salts and palladium from the first reaction step was key. Compatibility between palladium and enzyme catalysts can be a problem, as was the case for a halogenase‐Suzuki‐coupling cascade in batch reported by Latham *et al* (Scheme 6C).^[144]^ Using free enzyme, ultrafiltration or compartmentalization with a semi‐permeable membrane had to be used to physically separate the enzyme and Pd catalyst. Alternatively, immobilization of the halogenase into CLEAs was also successful.

**Figure 18:**
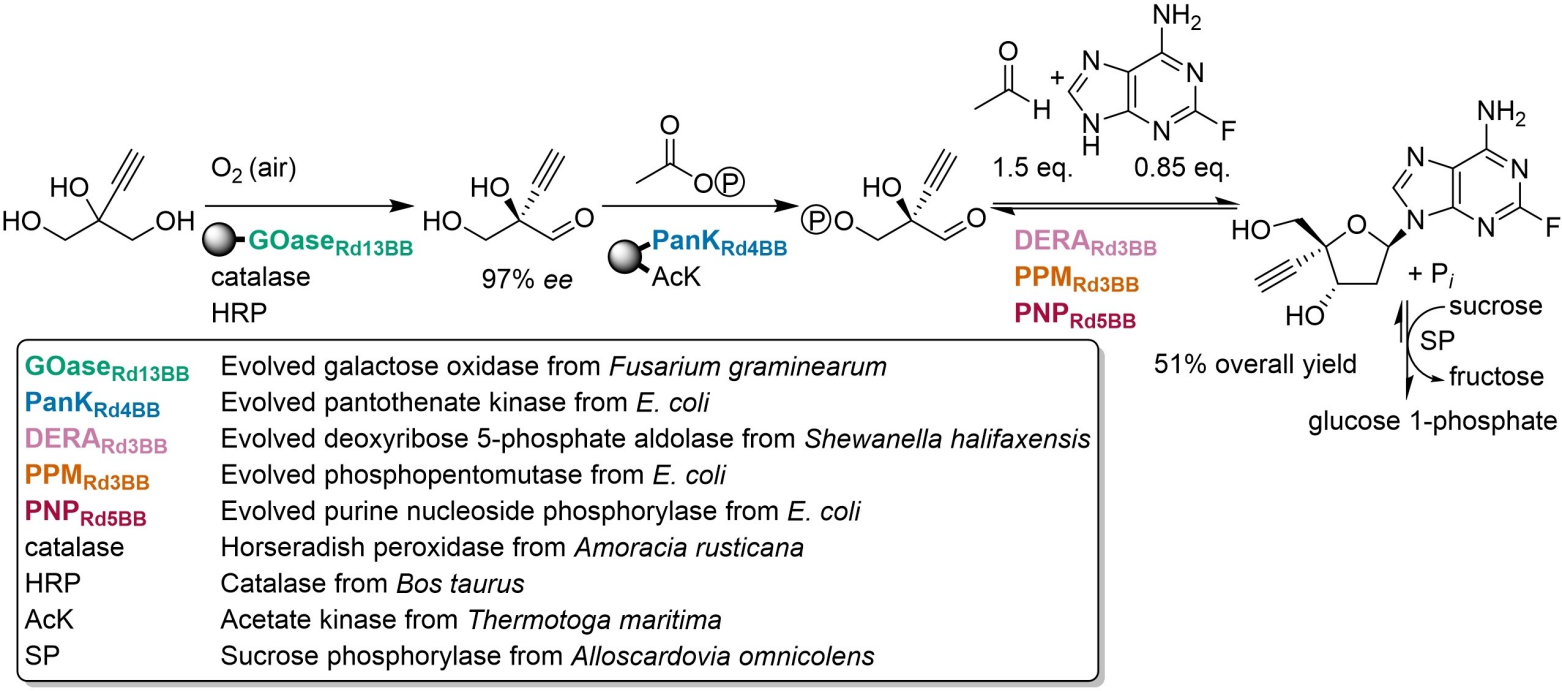
Nine‐enzyme cascade to produce the HIV drug islatravir. Five enzymes had to be evolved. Compared to a chemical synthesis, steps were reduced by more than half and yield was almost doubled. No purification of intermediates was necessary. Immobilized enzymes shown attached to spheres. Figure adapted from Huffman *et al*.^[145]^

In another collaboration between Codexis and Merck, a three‐step, nine‐enzyme cascade to synthesize the HIV drug islatravir was developed (Figure 18).^[145]^ This involved engineering of five enzymes to accept unnatural substrates, as well as enzyme immobilization to simplify the final purification. For this, a cost‐effective affinity immobilization using a His‐tag was chosen for the first two steps, while the last step used free enzymes. Enzymes from seven organisms were used, and each step involved enzymes from either two or three organisms and one or three evolved enzymes. Thus, by bringing together the right enzymes from the right organisms (a testament to the vast number of genome sequences that are available) and applying directed evolution only were needed, the number of steps in the synthesis of islatravir was cut more than in half (from 12–18 steps), and the overall yield was almost doubled (51 % vs 37 % previously reported^[146]^). Atom economy was improved, overall waste was reduced, and hazardous reagents (such as a Birch reduction in Fukuyama *et al*.’s synthesis^[146]^) were avoided.

## 8 Summary and Outlook

Through the elucidation of enzymatic mechanisms and enzyme structures, as well as the development of powerful tools for DNA manipulation, engineered enzymes are being applied in increasingly complex syntheses. However, challenges remain. Enzyme engineering is time consuming and while excellent enzyme variants have been created, it is not always successful. Even though significant advances have been made in the understanding of protein folding and predicting the effects of mutations, we still rely on the principles of directed evolution developed by Frances Arnold. Further research into the properties of enzymes is necessary to increase their predictability, which will lead to a future where enzymes can be applied more routinely.

Promising approaches include the use of increasingly sophisticated machine learning, based on large sets of sequences of known function (either wild‐type sequences or mutants).[^120^, ^147^] Exploring a large sequence space has become increasingly possible due to the advances in gene synthesis, building on DNA synthetic strategies and improvements in DNA sequencing mentioned earlier.^[148]^ Understanding the trajectories of substrates in addition to their interactions once inside the active site (the aim of traditional substrate docking) allows new target residues to be identified.^[149]^ Additionally, the *de‐novo* design of proteins has offered a window into entirely new protein sequences unknown in nature. In addition to offering exciting new catalysts, this is also an invaluable way of testing our understanding of protein folding and the factors that influence catalytic efficiency.^[150]^ Expanding the genetic code to include unnatural amino acids with functional groups not found in nature may further increase enzyme performance and open up new reactions currently outside of the scope of biocatalysis.^[151]^ Synthetic biology, which focusses on the introduction of new metabolic pathways into organisms to produce valuable chemicals from cheap and renewable starting materials, is another exciting emerging field.^[152]^

More fundamental challenges also remain, such as protein expression which is often difficult to predict. While *E. coli* has been undoubtedly the expression organism of choice, due to the vast number of molecular biology tools available and the ease with which it can be grown, some proteins cannot be expressed at high levels or in soluble form and, recently, concerns about low levels of endotoxins that are sufficient to cause an immune response have been raised.^[153]^ Thus, alternative expression systems are needed such as fungi and extremophilic archaea to allow for the expression of proteins incompatible with *E. coli* and bypass potential toxicity.^[154]^

Lastly, it is still extremely challenging to bring a biocatalytic process to market. Often, even heavily engineered enzymes fall short in terms of space‐time‐yield compared to the best heterogeneous catalysts (particularly challenging for bulk chemicals). Additionally, a significant investment of both time and money is necessary to develop a biocatalytic process. This is especially true for synthetic biology, e. g. in the case of the anti‐malarial drug artemisinin which required 10 years of research and >$150 million to engineer an organism that could produce it.^[155]^ Clearly, additional research to address these issues is needed and a closer interaction between academia and industry could further speed up the process. Nonetheless, the many examples of successful biocatalytic processes mentioned in this review (as well as many not mentioned) highlight the power of enzymes and the bright future of the field of biocatalysis.

## Conflict of interest

The authors declare no conflict of interest.

## Biographical Information

*Growing up in Germany, Christian M. Heckmann studied Chemistry at the University of Nottingham, earning his MSci in 2017. He then started a PhD with Francesca Paradisi in collaboration with Johnson Matthey, studying biocatalytic approaches to the synthesis of chiral amines*.

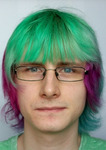

## Biographical Information

*Prof. Francesca Paradisi is the Chair of Pharmaceutical and Bioorganic Chemistry at the University of Bern. Biocatalysis as a sustainable approach to synthesis of valuable products is the focus of her research group. In particular, the group developed a number of enzyme‐based processes in continuous flow, reducing the gap between academic discovery and industrial application*.